# Molecular Characterization of α- and β-Thalassaemia Among Children From 1 to 10 Years of Age in Guangxi, A Multi-Ethnic Region in Southern China

**DOI:** 10.3389/fped.2021.724196

**Published:** 2021-08-23

**Authors:** Sheng He, Dongming Li, Shang Yi, Xiuning Huang, Chaofan Zhou, Biyan Chen, Yangjin Zuo, Li Lin, Faqin Chen, Hongwei Wei

**Affiliations:** ^1^Key Laboratory of Basic Research for Guangxi Birth Defects Control and Prevention, Guangxi Zhuang Autonomous Region Women and Children Care Hospital, Nanning, China; ^2^Department of Laboratory Medicine, Youjiang Medical University for Nationalities, Baise, China

**Keywords:** thalassemia, prevalence and spectrum, children, Guangxi, China

## Abstract

**Background:** Thalassemia is one of the most common genetic diseases in southern China. Howerver, population in different regions or different population has their own spectrums of thalassemia. To investigate the prevalence and spectrum features of thalassemia among children in Guangxi. Hematology and genetic analysis were performed on 71,459 children aged 1–10 years in various regions of Guangxi.

**Results:** A total of 11,821 children were diagnoses with thalassemia including 7,615 (10.66%) subjects of α-thalassemia, 3,507 (4.90%) subjects of β-thalassemia, and 699 (0.98%) cases with both α- and β-thalassemia. Nine α-thalassemia mutations and 30 genotypes were identified among the α-thalassemia children. The - -^SEA^ and - -^SEA^/αα were the most frequent mutation and genotype, respectively. One α-thalassemia fusion gene and a rare 2.4 kb deletion both causing α^+^-thalassemia were identified, respectively. Thirteen β-thalassemia mutations and 31 genotypes were characterized among the β-thalassemia children, with the most common mutation CD41-42 (-CTTT) accounting for 46.05% of the β-mutations. Two rare mutations IVS-II-5 (G>C), and IVS-I-2 (T>C) were firstly identified. Furthermore, 92 genotypes were identified among 699 children with both α- and β-thalassemia.

**Conclusions:** Our findings highlight the great heterogeneity and the extensive spectrum of thalassemia among children in Guangxi, which provide an available reference for prevention of thalassemia in this area.

## Introduction

The thalassemias are a group of inherited hemoglobin disorders characterized by microcytic hypochromic anemia resulting from reduced or absent synthesis of one or more of the globin chains of hemoglobin (1). According to the type of globin involved, thalassemias are divided into α-, β-, δ-thalassemia, with α- and β-thalassemia most widely distributed worldwide (2). Clinical phenotype of people with thalassemia varies from almost asymptomatic to a lethal hemolytic anemia. Carriers of heterozygous globin gene mutations or patients with mild thalassaemia may not have symptoms of anemia. However, patients with severe α-thalassemia (i.e., hydrops fetalis) always die *in utero* or soon after birth and sometimes leading to the mortality of the pregnant mother (3, 4). β-thalassemia major patients have a severe form of anemia requiring lifelong transfusion and may have shortened life span (3). To date, there is no effective treatment for patients with thalassemia major, except bone marrow transplantation (2). Thalassemia major has caused a huge burden on society and seriously affected the quality of life of people in developing countries (3, 5). Therefore, it is critical to implement prenatal diagnosis and genetic counseling to prevent the birth of children with thalassemia major on the basis of grasping the molecular epidemiological characteristics of the frequency and distribution of thalassemia.

Approximately 5% of the global population are carriers of thalassemia (2, 6). Thalassemias are particularly frequent in Mediterranean countries, the Middle East, Africa, and Southeast Asia (2). In China, thalassemia is widely distributed in the southern bank of the Yangtze River, particularly in the three southern provinces of Guangdong, Guangxi, and Jiangxi (7–9). Previous studies showed that the frequency of thalassemia reached about 20% in Guangxi province, a multi-ethnic region. However, these studies only focused on the adult population, and there has been no report about the spectrum thalassemia among children (8, 10, 11). Since the spectrum, and frequencies of α- and β-thalassaemia alleles vary considerably with geographic locations and different populations (12), this study was performed to characterize α- and β-thalassaemia mutations at the molecular level among children in Guangxi province.

## Materials and Methods

### Human Subjects

This study population included 71,459 aged from 1 to 10-year-old children in difference regions of Guangxi for hemoglobinopathy screening between January 1, 2011 and December 31, 2019. These subjects visited medical units for routine healthy examination including blood tests, and the discarded blood samples were used for further study. Information records of nationality, sex, age, dialect, Guangxi aborigines or not and written consent forms were available in Chinese to ensure comprehensive understanding of the study objectives. Informed consent was signed by the participants or their guardians. All studies were approved by the Ethic Committee of Guangxi Zhuang Autonomous Region Women and Children Care Hospital. All procedures were carried out in accordance with ethical guidelines for human subject's research.

### Hematological Analysis

**Figure 1:**
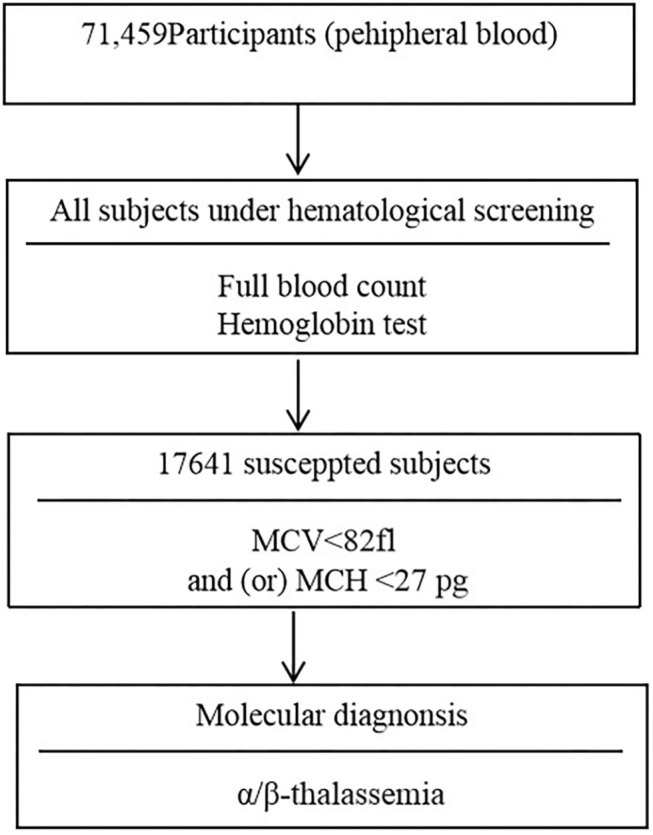
Diagram for the screening of α/β-thalassemia.

Two ml volume of peripheral venous blood samples were collected into an EDTA anticoagulated tube. Peripheral blood counts and red blood cell incidences were determined using a SYSMEX XE800i automatic blood cell analyzer (Kobe, Japan). Hemoglobin electrophoresis was applied to analyze the percentage of hemoglobin Hbs A, A_2_, F, and any abnormal hemoglobin variant. The children with microytosis [low mean corpuscular volume (MCV) values (<82 fl) and/or low mean cell hemoglobin (MCH) values (<27 pg)] after the exclusion of iron deficiency anemia were consider suspected thalassemia children. Moreover, children with low HbA_2_ (<2.5%) and high HbA_2_ (≥3.5%) values were considered possible α-thalassemia carriers and β-thalassemia carriers, respectively (7, 13). The flowchart of screening strategy used in this study is illustrated in Figure 1.

### Genetic Analysis

Genomic DNA of subjects with microcytosis were isolated using a DNA blood extraction kit (Tiangen Bio-Tech Co. Ltd., Beijing, China) according to the manufacturer's instructions. The four known α-thalassemia deletionas [- -^SEA^ (NG_000006.1:g.26264_45564del19301), -α^3.7^ (NG_000006.1:g.34164_37967del3804), -α^4.2^ (AF221717), - -^THAI^ (NG_000006.1:g/10664_44164del33501)], three α-thalassemia mutations [Hb Constant Spring, Hb CS (*HBA2*:c.427T>C), Hb Quong Sze (Hb QS, *HBA2*:c.377T>C), and Hb Westmead (Hb WS, *HBA2*:c.369C>G)] and 17 known β-thalassemia mutations most commonly seen in the Chineses population [CD41-42(-TCTT) (*HBB*:c.126_129delCTTT), IVS-II-654(C → T) (*HBB*:c.316-197C>T), CD17(A → T) (*HBB*:c.52A>T),−28(A → G) (*HBB*:c.-78A>G), CD26 (G → A) (*HBB*:c.79G>A), CD71-72 (+A), (*HBB*:c.216_217insA), CD43 (G → T) (*HBB*:c.130G>T),−29 (A → G) (*HBB*:c.-79A>G), CD14-15 (+G) (*HBB*:c.45_46insG), CD27-28 (+ C) (*HBB*:c.84_85insC),−32 (C → A) (*HBB*:c.-82C>A),−30 (T → C) (*HBB*:c.-80T>C), IVS-I-1(G → T) (*HBB*:c.92+1G>T), IVS-I-5 (G → C) (*HBB*:c.92+5G>A), CD31 (-C) (*HBB*:c.94delC), and CapM (-AAAC) (*HBB*:c.-50A>C) were identified as described by He et al. (13). Multiplex ligation-dependent probe amplification (MLPA) (Service XS^TM^ Leiden, the Netherlands) was performed to detect gross deletions in the α- or β-globin gene cluster in case of new mutations. DNA sequencing was applied to detect unknown and rare globin mutations.

### Statistical Analysis

Statistical analysis was conducted with SSPS 17.0 software. The prevalence of different thalassemia alleles was calculated from modified Hardy-Weinberg formula.

## Results

### The Characteristic of Thalassemia Among Children in Guangxi Province

From 2011 to 2019, a total of 17,641 (24.69%) children with microcytosis were found. 11,821 out of the 17,641 suspected children were diagnosed with thalassemia, including 7,615 (10.64%) subjects of α-thalassemia, 3,507 (4.90%) subjects of β-thalassemia and 699 (0.98%) subjects with both α- and β-thalassemia. Among the 7,615 α-thalassemia children, 6,734 (88.43%) cases had heterozygote mutations and 881 cases (11.57%) were with homozygote and compound heterozygote mutations. The - -^SEA^/αα was the most frequent genotype, accounting for more than half of all α-thalassemia genotypes (53.51%). The other most common genotypes of α-thalassemia were - α^3.7^/αα, α^CS^α/αα, - α^4.2^/αα, --^SEA^/-α^3.7^, and α^WS^α/αα (Table 1).

**Table 1:** Distribution of genotypes among the 7,615 α-thalassemia children in Guangxi.

**Genotype**	**Number**	**Phenotype**	**Frequency (%)**
Heterozygote	6,734		88.43
--^SEA^/αα	4,075	α^0^/α	53.51
-α^3.7^/αα	1,157	α^+^/α	15.19
α^CS^α/αα	622	α^+^/α	8.17
-α^4.2^/αα	488	α^+^/α	6.41
α^WS^α/αα	250	α^+^/α	3.28
α^QS^α/αα	88	α^+^/α	1.16
--^THAI^/αα	53	α^0^/α	0.70
-α^2.4^/αα	1	α^+^/α	0.01
Compound heterozygote	799		10.49
--^SEA^/-α^3.7^	281	α^0^/α^+^	3.69
--^SEA^/-α^4.2^	143	α^0^/α^+^	1.88
--^SEA^/α^CS^α	129	α^0^/α^+^	1.69
--^SEA^/α^WS^α	72	α^0^/α^+^	0.95
-α^3.7^/α^CS^α	40	α^+^/α^+^	0.53
-α^3.7^/-α^4.2^	31	α^+^/α^+^	0.41
-α^3.7^/α^WS^α	26	α^+^/α^+^	0.34
--^SEA^/α^QS^α	17	α^0^/α^+^	0.22
-α^4.2^/α^CS^α	14	α^+^/α^+^	0.18
-α^4.2^/α^WS^α	12	α^+^/α^+^	0.16
α^WS^α/α^CS^α	11	α^+^/α^+^	0.15
-α^3.7^/α^QS^α	7	α^+^/α^+^	0.09
α^WS^α/α^QS^α	6	α^+^/α^+^	0.08
--^THAI^ /-α^3.7^	5	α^0^/α^+^	0.07
-α^4.2^/α^QS^α	1	α^+^/α^+^	0.01
--^THAI^/-α^4.2^	1	α^0^/α^+^	0.01
--^THAI^/α^WS^α	2	α^0^/α^+^	0.03
--^THAI^ αfusion gene	1	α^0^/α^+^	0.01
Homozygote	82		1.08
-α^3.7^/-α^3.7^	48	α^+^/α^+^	0.63
α^CS^α/α^CS^α	14	α^+^/α^+^	0.18
-α^4.2^/-α^4.2^	13	α^+^/α^+^	0.17
α^WS^α/α^WS^α	7	α^+^/α^+^	0.09
Total	7,615		100.00

*α, normal production of the α-globin polypeptide chain; α0, no production of the α-globin polypeptide chain; α+, reduced production of the α-globin polypeptide chain*.

### Distribution of Thalassemia Genotypes Among Children

Thirty-five genotypes were found in 3,507 β-thalassemia children, including 3,419 heterozygotes, 31 mutant homozygotes, and 57 compound heterozygotes, accounting for 97.49, 0.88, and 1.63%, respectively (Table 2). β^CD41−42^/β^N^ was the most prevalent genotype, accounting for 45.11% of all β-thalassemia genotypes. Most of the remaining genotypes were β^CD17^/β^N^, β^IVS−II−654^/β^N^, β^CD71−72^/β^N^, β^−28^/β^N^, β^CD26^/β^N^, β^IVS−I−1^/β^N^, and β^CD43^/β^N^. Overall, these eight genotypes accounted for 96.72% of all β-thalassemia genotypes (Table 2). Six hundred and ninety nine children with 92 genotypes carried both α and β-globin mutations. Among these children, four top frequent types were --^SEA^ /αα combined with β^CD41−42^/β^N^ (17.60%), –α^3.7^/αα combined with β^CD41−42^/β^N^ (9.88%), –^SEA^/αα combined with β^CD17^/β^N^ (9.02%), and - α^3.7^/αα combined with β^CD17^/β^N^ (7.30%) (Supplementary Table 1).

**Table 2:** Distribution of genotypes among the 3,507 β-thalassemia children in Guangxi.

**Genotype**	**Cases**	**Phenotype**	**Frequency (%)**
Heterozygote	3,419		97.49
β^CD41−42^/β^N^	1,582	β^0^/β^N^	45.11
β^CD17^/β^N^	974	β^0^/β^N^	27.77
β^IVS−II−654^/β^N^	248	β^0^/β^N^	7.07
β^CD71−72^/β^N^	160	β^0^/β^N^	4.56
β^−28^/β^N^	157	β^+^/β^N^	4.48
β^CD26^/β^N^	145	β^0^/β^N^	4.14
β^IVS−I−1^/β^N^	79	β^0^/β^N^	2.25
β^CD43^/β^N^	47	β^0^/β^N^	1.34
β^−29^/β^N^	10	β^+^/β^N^	0.28
β^27−28^/β^N^	10	β^0^/β^N^	0.28
β^CD14−15^/β^N^	4	β^0^/β^N^	0.11
β^VS−II−5^/β^N^	1	β^+^/β^N^	0.03
β^VS−I−5^/β^N^	1	β^+^/β^N^	0.03
β^CAP+1^/β^N^	1	β^+^/β^N^	0.03
Homozygote	31		0.88
β^CD41−42^/β^CD41−42^	19	β^0^/β^0^	0.54
β^CD17^/β^CD17^	9	β^0^/β^0^	0.26
β^IVS−I−1^/β^IVS−I−1^	3	β^0^/β^0^	0.09
Compound heterozygote	57		1.63
β^CD41−42^/β^CD17^	14	β^0^/β^0^	0.40
β^CD41−42^/β^−28^	7	β^0^/β^+^	0.20
β^41−42^/β^CD26^	5	β^0^/β^+^	0.14
β^CD41−42^/β^IVS−II−654^	4	β^0^/β^+^	0.11
β^CD17/^β^−28^	4	β^0^/β^+^	0.11
β^CD71−72^/β^CD17^	4	β^0^/β^0^	0.11
β^CD41−42^/β^IVS−I−1^	4	β^+^/β^0^	0.11
β^CD17/^β^CD26^	3	β^0^β^0^	0.09
β^IVS−II−654^/β^CD17^	3	β^0^/β^0^	0.09
β^CD71−72^/β^CD26^	1	β^0^/β^+^	0.03
β^CD17^/β^IVS−I−1^	1	β^0^/β^0^	0.03
β^CD43^/β^CD17^	1	β^+^/β^0^	0.03
β^CD41−42^/β^CD71−72^	1	β^0^/β^0^	0.03
β^CD41−42^/β^CD27/28^	1	β^0^/β^+^	0.03
β^IVS−II−654^/β^CD26^	1	β^0^/β^+^	0.03
β^IVS−II−654^/ββ^IVS−II−5^	1	β^0^/β^+^	0.03
β^CD41−42^/β^CAP^	1	β^0^/β^+^	0.03
β^−28^/β^CD26^	1	β^0^/β^+^	0.03
Total	3,507		100.00

*β^N^, normal production of the β-globin polypeptide chain; β^0^, no production of the β-globin polypeptide chain; β^+^, impaired production of the β-globin polypeptide chain*.

### Allele Frequency of α-Thalassemia and β-Thalassemia Among Children

Tables 3, 4 showed the frequency of a specific type of mutation in all α (or β) mutant chromosomes in thalassemia children. Twenty-four mutations were identified including nine alpha gene mutations and 15 beta gene mutations. The - -^SEA^ was the most frequent mutation, accounting for 54.23% of all α mutant chromosomes. The other high frequency mutations were -α^3.7^, α^CS^α, α^WS^ α, and -α^4.2^, with allele frequencies of 19.76, 9.92, 8.58, and 5.40%, respectively (Table 3). Two rare mutations: a fusion and -α^2.4^ were identified in these children. Of β-globin mutant chromosomes, eight mutations accounted for as much as 99.22% of all β-thalassemia defects. These mutations in the order of frequency were CD41-42 (-CTTT) (46.07%), CD17 (A>T) (28.44%), IVS-II-654(C>T) (7.10%),−28 (A>G) (4.84%), CD26 (Hb E) (G>A) (4.58%), CD71-72 (+A) (4.23%), IVS-I-1 (G>T) (2.66%), and CD43 (G>T) (1.30%). The other seven mutations with frequencies of no more than 1% were−29 (A>G), CD27-28 (+ C), CD14-15 (+G), IVS-II-5 (G>C), CAP (A>C), IVS-I-5 (G>C), IVS-I-2 (T>C) (Table 4).

**Table 3:** Allele frequency of α-thalassemia among the 71,459 children in Guangxi.

**Mutation**	**HGVS name**	**Type**	**Allele (*n*)**	**Frequency (%)**
--^SEA^	NG_000006.1:g.26264_ 45564del19301	α^0^	5,008	54.23
-α^3.7^	NG_000006.1:g.34164_ 37967del3804	α^+^	1,825	19.76
α^CS^α	*HBA2*:c.427T>C	α^+^	916	9.92
-α^4.2^	AF221717	α^+^	792	8.58
α^WS^α	*HBA2*:c.369C>G	α^+^	499	5.40
α^QS^α	*HBA2*:c.377T>C	α^+^	127	1.38
--^THAI^	NG_000006.1:g/10664_441 64del33501	α^0^	66	0.71
-α^2.4^	*HBA1*: g36859_39252del2392	α^+^	1	0.01
α^fusion^		α^+^	1	0.01
Total			9,235	100.00

*α, normal production of the α-globin polypeptide chain; α^0^, no production of the α-globin polypeptide chain; α^+^, reduced production of the α-globin polypeptide chain*.

**Table 4:** Allele frequency of β-thalassemia among the 71,459 children in Guangxi.

**Mutation**	**HGVS name**	**Type**	**Allele (*n*)**	**Frequency (%)**
CD41-42 (-TTCT)	*HBB*:c.126_129delCTTT	β^0^	1,991	46.07
CD17 (A>T)	*HBB*:c.52A>T	β^0^	1,229	28.44
IVS-II-654 (C>T)	*HBB*:c.316-197C>T	β^+^	307	7.10
−28 (A>G)	*HBB*:c.-78A>G	β^+^	209	4.84
CD26 (G>A)	*HBB*:c.79G>A	β^+^	198	4.58
CD71-72 (+A)	*HBB*:c.216_217insA	β^0^	183	4.23
IVS-I-1 (G>T)	*HBB*:c.92+1G>T	β^0^	115	2.66
CD43 (G>T)	*HBB*:c.130G>T	β^0^	56	1.30
−29 (A>G)	*HBB*:c.-79A>G	β^+^	13	0.30
CD27-28 (+C)	*HBB*:c.84_85insC	β^0^	11	0.25
CD14-15 (+G)	*HBB*:c.45_46insG	β^0^	4	0.09
IVS-II-5 (G>C)	*HBB*:c.315+5G>C	β^0^	2	0.05
CAP (A>C)	*HBB*:c.-50A>C	β^+^	2	0.05
IVS-I-5 (G>A)	*HBB*:c.92+5G>A	β^+^	1	0.02
IVS-I-2 (T>C)	*HBD*:c.92+2T>C	(deltabeta) zero	1	0.02
Total			4,322	100.00

*β^N^, normal production of the β-globin polypeptide chain; β^0^, no production of the β-globin polypeptide chain; β^+^, impaired production of the β-globin polypeptide chain*.

## Discussion

In the present study, we have firstly reported the prevalence of thalassemia at molecular level and the related mutation among children in Guangxi, a southwestern province in China. The data indicated there was a high prevalence of thalassemia among children in this region. The prevalence of α-, β-, and α + β-thalassemia was 10.64, 4.90, and 0.98%, respectively. The overall prevalence was 16.52%. It is was slightly lower than the average level of Guangxi province (19.52%) (8). The difference may be due to the population and the strategy selected. Previous studies were based on epidemiological surveys in mixed populations including neonates, children, adults, and pregnant women (8, 10, 11). Whereas, the research subjects in our study were only focused on the children from 1 to 10-year-old in various regions of the province and can more objectively reflect the rate among the general children. Traditional methods using hematological test as a primary screening for thalassemia is a limitation in our study. Some silent thalassemia carriers with normal or borderline red cell indices and/or HbA_2_ levels might be missed and were not detected (14, 15). Recently, screening for both α- and β-thalassemia genes by next-generation sequencing (NGS) has been introduced. Limitations of our study, such as possible missed carriers during primary screening will be further addressed in the future (15, 16). When compared with the overall prevalence with other regions in China, the frequencies of thalassemia was higher than that in Guangdong (11.07%) (8), Yunnan (9.7%) (17), Fujian (4.57%) (18), Sichuan (3.43%) (19), and Jiangxi province (9.49%) (9) of China.

Previous studies have shown that α-thalassemia is mainly caused by three types of gene deletions (--^SEA^, -α^3.7^, and -α^4.2^) and two types of gene non-deletion mutations (α^CS^α and α^WS^α) in Chinese population (7, 8, 16). In this study, five deletions and 3 non-deletions with 26 genotypes were identified among α-thalassemia children. The - -^SEA^ and - -^SEA^/αα were the most frequent α-thalassemia mutation and genotype, respectively, which is consistent with the observations in Guangxi adult population and Yunnan children (8, 14). In addition, the Thailand deletion (--^THAI^) which was previously described in Southeast Asian and Taiwan aboriginals (20, 21), and be regarded as rare mutation in mainland China was identified in 65 children, with the allele frequency of 0.7% in our study, which indicates that it should be considered a common mutation. Moreover, we identified an α-thalassemia fusion gene in this study. This fusion gene was formed by the fusion of α2 and Ψα1 sequence of α-globin gene, which changed the 3′UTR of the α2 gene and gave rise to a polyadenylation signal mutation. A polyadenylation signal mutation could produce an extensive transcript of the α2 gene and cause α^+^-thalassemia (22), which was confirmed by the hematology parameters of the child. These findings enriched the gene mutation database of thalassemia among children in Guangxi province and might have great significance for genetic counseling, thalassemia genetic diagnosis, and prevention.

In contrast of α-thalassemia, most cases of β-thalassemia are caused by point mutations which lead to a reduction (β^+^) or complete absence (β^0^) of β-globin chain synthesis from the affected allele (23). At present, more than 300 mutations in the β-globin gene have been characterized world-wide (15). In China, 17 common and more than 23 rare β-globin gene mutations have been found, while the type frequency and spectrum of these mutations vary considerably with geographical location and different populations (9). In this study, 13 mutations with 35 genotypes were identified in the 3,507 β-thalassemia children. CD41-42(-TTCT) is the most frequent β-thalassemia mutation with an allele frequency of 46.07%, which is similar to the situation reported in other regions such as the Guilin Region (52.02%) (24), Guangxi Province (48.37%) (11), Guangdong province (36.4%) (7). Whereas, it was different from Guizhou province, Meizhou city of Guangdong Province, and Yunnan province (17, 25, 26). CD17 (A>T) was the most frequent β-thalassemia mutation in Baise Region and Guizhou province (13, 25). IVS-II-654 and CD26 (β^E^) are the principal mutations of β-thalassemia in Meizhou city of Guangdong Province (26), and in Yunnan region, respectively [18]. Additionally, two rare mutations IVS-II-5 (G>C) and IVS-I-2 (T>C) were identified in the children, which was the first report of the abnormal hemoglobin in the Chinese population according to our knowledge. The distribution of the mutation spectrum with the geographical locations reflected the heterogeneity of β-thalassemia and highlighted the screen for beta thalassemia in the different populations and locations.

Ninety-two genotypes were identified among 699 children with both α- and β-thalassemia, which indicates the molecular background of α and β-thalassemia mutation among the children in Guangxi is more complex and extensive heterogeneity than that in other areas of China. Guangxi is a multi-ethnic province, where nearly 40 different ethnic groups live, thus the heterogeneity may be caused by population admixture, marriage patterns, and migrations. With a better knowledge of the thalassemia mutations present in different populations, it may be possible to develop a more rational approach to population screening for control and counseling of disease in an area (7). Our study has analyzed mutations among children that have shown patterns in Guangxi which can be used for quicker and more convenient identification of mutations while conducting newborn screening and prenatal diagnosis, and provide dates for a large population screening plan of thalassemia in Guangxi province.

## Conclusion

In conclusion, our study has demonstrated the great heterogeneity and the extensive spectrum of α-thalassemia and β-thalassemia mutations among children in Guangxi. The findings provide the valuable information for pre-marital and pre-pregnancy screening, prenatal diagnostic services, and designing appropriate prevention programs to control the incidence of severe thalassemia in this area.

## Data Availability Statement

The original contributions presented in the study are included in the article/Supplementary Material, further inquiries can be directed to the corresponding authors.

## Ethics Statement

The study was approved by the Ethic Committee of Guangxi Zhuang Autonomous Region Women and Children Care Hospital. The patients/participants provided their written informed consent to participate in this study.

## Author Contributions

SH and DL conceived the experiments and wrote the manuscript. SY and XH performed data analyzed and summarized results performed. CZ and BC collected the data. YZ and LL performed part of genetic tests. FC and HW conceived the idea of the study. All authors have read and approved the manuscript.

## Conflict of Interest

The authors declare that the research was conducted in the absence of any commercial or financial relationships that could be construed as a potential conflict of interest.

## Publisher's Note

All claims expressed in this article are solely those of the authors and do not necessarily represent those of their affiliated organizations, or those of the publisher, the editors and the reviewers. Any product that may be evaluated in this article, or claim that may be made by its manufacturer, is not guaranteed or endorsed by the publisher.
