# Supplementary material for: Molecular Characterization of α- and β-Thalassaemia Among Children From 1 to 10 Years of Age in Guangxi, A Multi-Ethnic Region in Southern China
Source: Front Pediatr. 2021 Aug 23;9:724196. doi: 10.3389/fped.2021.724196 (PMC8419341; doi:10.3389/fped.2021.724196)
Supplement: Supplementary file 1 [file Data_Sheet_1.PDF]

Supplement Table 1

Distribution of α- compound β-thalassemia genotypes among the 71,459 children in Guangxi.

Genotype	cases	Frequency (%)
$--^{SEA}/\alpha\alpha$、 $\beta^{CD41-42}/\beta^{N}$	123	17.6
$-\alpha^{3.7}/\alpha\alpha$、 $\beta^{CD41-42}/\beta^{N}$	69	9.87
$--^{SEA}/\alpha\alpha$、 β^{CD17}/β^{N}	63	9.01
$-\alpha^{3.7}/\alpha\alpha$、 β^{CD17}/β^{N}	51	7.30
$\alpha^{WS}\alpha/\alpha\alpha$、 $\beta^{CD41-42}/\beta^{N}$	46	6.58
$\alpha^{CS}\alpha/\alpha\alpha$、 $\beta^{CD41-42}/\beta^{N}$	27	3.86
$\alpha^{WS}\alpha/\alpha\alpha$、 β^{CD17}/β^{N}	24	3.43
$-\alpha^{4.2}/\alpha\alpha$、 $\beta^{CD41-42}/\beta^{N}$	22	3.15
$--^{SEA}/\alpha\alpha$、 β^{CD26}/β^{N}	21	3.00
$\alpha^{CS}\alpha/\alpha\alpha$、 β^{CD17}/β^{N}	21	3.00
$-\alpha^{4.2}/\alpha\alpha$、 β^{CD17}/β^{N}	20	2.86
$--^{SEA}/\alpha\alpha$、 β^{-28}/β^{N}	18	2.58
$--^{SEA}/\alpha\alpha$、 $\beta^{IVS-II-654}/\beta^{N}$	15	2.15
$-\alpha^{3.7}/\alpha\alpha$、 $\beta^{IVS-II-654}/\beta^{N}$	10	1.43
$--^{SEA}/\alpha\alpha$、 $\beta^{IVS-I-1}/\beta^{N}$	8	1.14
$\alpha^{WS}/\alpha\alpha$、 $\beta^{IVS-II-654}/\beta^{N}$	8	1.14
$-\alpha^{3.7}/\alpha\alpha$、 $\beta^{-28}/\beta N$	8	1.14
$\alpha^{CS}/\alpha\alpha$、 $\beta^{IVS-II-654}/\beta^{N}$	7	1.00
$\alpha^{WS}\alpha/\alpha\alpha$、 $\beta^{IVS-I-1}/\beta^{N}$	6	0.86
$--^{SEA}/\alpha^{3.7}$、 $\beta^{CD41-42}/\beta^{N}$	6	0.86
$--^{SEA}/\alpha^{3.7}$、 β^{CD17}/β^{N}	6	0.86
$--^{SEA}/\alpha\alpha$、 $\beta^{CD71-72}/\beta^{N}$	5	0.72
$--^{SEA}/\alpha\alpha$、 $\beta^{CD41-42}/\beta^{CD41-42}$	5	0.72
$-\alpha^{4.2}/\alpha\alpha$、 β^{CD26}/β^{N}	5	0.72
$-\alpha^{4.2}/\alpha\alpha$、 β^{-28}/β^{N}	5	0.72
$-\alpha^{3.7}/\alpha\alpha$、 β^{CD26}/β^{N}	5	0.72
$-\alpha^{3.7}/\alpha\alpha$、 β^{CD43}/β^{N}	5	0.72
$-\alpha^{4.2}/\alpha\alpha$、 $\beta^{IVS-II-654}/\beta^{N}$	4	0.58
$-\alpha^{3.7}/\alpha\alpha$、 $\beta^{IVS-I-1}/\beta^{N}$	4	0.58
$-\alpha^{3.7}/\alpha\alpha$、 $\beta^{CD71-72}/\beta^{N}$	4	0.58
$\alpha^{WS}\alpha/\alpha\alpha$、 β^{CD26}/β^{N}	3	0.43
$\alpha^{WS}\alpha/\alpha\alpha$、 $\beta^{CD41-42}/\beta^{CD17}$	3	0.43
$\alpha^{WS}/\alpha\alpha$、 $\beta^{CD41-42}/\beta^{IVS-II-654}$	3	0.43
$--^{SEA}/\alpha\alpha$、 β^{-29}/β^{N}	2	0.29
$\alpha^{WS}\alpha/\alpha\alpha$、 $\beta^{CD71-72}/\beta^{N}$	2	0.29
$\alpha^{WS}\alpha/\alpha\alpha$、 $\beta^{CD41-42}/\beta^{IVS-I-1}$	2	0.29
$\alpha^{QS}\alpha/\alpha\alpha$、 β^{CD26}/β^{N}	2	0.29
$\alpha^{QS}\alpha/\alpha\alpha$、 $\beta^{CD41-42}/\beta^{N}$	2	0.29

$\alpha^{CS}\alpha/\alpha\alpha$、$\beta^{CD71-72}/\beta^{N}$	2	0.29
$\alpha^{CS}\alpha/\alpha\alpha$、$\beta^{-28}/\beta^{N}$	2	0.29
$--^{SEA}/\alpha^{CS}\alpha$、$\beta^{CD41-42}/\beta^{N}$	2	0.29
$-\alpha^{4.2}/\alpha\alpha$、$\beta^{CD71-72}/\beta^{N}$	2	0.29
$--^{SEA}/-\alpha^{3.7}$、β^{-28}/β^{N}	2	0.29
$--^{THAI}/\alpha\alpha$、$\beta^{IVS-I-1}/\beta^{N}$	1	0.14
$--^{THAI}/\alpha\alpha$、$\beta^{CD41-42}/\beta^{N}$	1	0.14
$--^{THAI}/\alpha\alpha$、β^{-28}/β^{N}	1	0.14
$\alpha\alpha/--^{THAI}$、β^{CD17}/β^{N}	1	0.14
$--^{SEA}/\alpha\alpha$、$\beta^{CD71-72}/\beta^{CD17}$	1	0.14
$--^{SEA}/\alpha\alpha$、β^{CD43}/β^{N}	1	0.14
$--^{SEA}/\alpha\alpha$、$\beta^{CD43}/\beta^{CD17}$	1	0.14
$--^{SEA}/\alpha\alpha$、$\beta^{CD41-42}/\beta^{CD17}$	1	0.14
$\alpha^{WS}\alpha/\alpha\alpha$、$\beta^{CD17}/\beta^{CD26}$	1	0.14
$\alpha^{WS}\alpha/\alpha\alpha$、$\beta^{CD17}/\beta^{-28}$	1	0.14
$\alpha^{WS}\alpha/-\alpha^{4.2}$、$\beta^{CD26}/\beta^{N}$	1	0.14
$\alpha^{WS}\alpha/-\alpha^{3.7}$、$\beta^{CD41-42}/\beta^{N}$	1	0.14
$\alpha^{WS}\alpha/-\alpha^{3.7}$、$\beta^{CD41-42}/\beta^{-28}$	1	0.14
$\alpha^{WS}\alpha/-\alpha^{3.7}$、$\beta^{CD17}/\beta^{N}$	1	0.14
$--^{SEA}/\alpha^{WS}\alpha$、$\beta^{CD41-42}/\beta^{N}$	1	0.14
$--^{SEA/}\alpha^{WS}\alpha$、$\beta^{CD17}/\beta^{N}$	1	0.14
$\alpha^{QS}\alpha/\alpha\alpha$、$\beta^{-28}/\beta N$	1	0.14
$\alpha^{QS}\alpha/\alpha\alpha$、$\beta^{CD17}/\beta^{CD17}$	1	0.14
$--^{SEA}/\alpha^{QS}\alpha$、$\beta^{CD41-42}/\beta^{N}$	1	0.14
$--^{SEA}/\alpha^{QS}\alpha$、$\beta^{CD17}/\beta^{N}$	1	0.14
$\alpha^{CS}\alpha/\alpha\alpha$、$\beta^{IVS-I-2}/\beta^{N}$	1	0.14
$\alpha^{CS}\alpha/\alpha\alpha$、$\beta^{IVS-I-1}/\beta^{N}$	1	0.14
$\alpha^{CS}\alpha/\alpha\alpha$、$\beta^{CD41-42}/\beta^{IVS-I-1}$	1	0.14
$\alpha^{CS}\alpha/\alpha\alpha$、$\beta^{CD41-42}/\beta^{CD17}$	1	0.14
$\alpha^{CS}\alpha/\alpha\alpha$、$\beta^{-29}/\beta N$	1	0.14
$\alpha^{CS}\alpha/\alpha^{WS}\alpha$、$\beta^{CD41-42}/\beta^{N}$	1	0.14
$\alpha^{CS}\alpha/-\alpha^{4.2}$、$\beta^{CD26}/\beta^{N}$	1	0.14
$\alpha^{CS}\alpha/-\alpha^{4.2}$、$\beta^{CD41-42}/\beta^{N}$	1	0.14
$\alpha^{CS}\alpha/-\alpha^{3.7}$、$\beta^{CD41-42}/\beta^{N}$	1	0.14
$--^{SEA}/\alpha^{CS}\alpha$、$\beta^{CD26}/\beta^{N}$	1	0.14
$--^{SEA}/\alpha^{CS}\alpha$、$\beta^{CD17}/\beta^{N}$	1	0.14
$-\alpha^{4.2}/\alpha\alpha$、$\beta^{IVS-I-1}/\beta^{N}$	1	0.14
$-\alpha^{4.2}/\alpha\alpha$、$\beta^{CD43}/\beta^{N}$	1	0.14
$-\alpha^{4.2}/\alpha\alpha$、$\beta^{CD41-42}/\beta^{CD26}$	1	0.14
$-\alpha^{4.2}/\alpha\alpha$、$\beta^{CD41-42}/\beta^{CD41-42}$	1	0.14
$-\alpha^{4.2}/\alpha\alpha$、$\beta^{CD41-42}/\beta^{CD17}$	1	0.14
$-\alpha^{4.2}/\alpha\alpha$、$\beta^{CD17}/\beta^{CD17}$	1	0.14
$-\alpha^{4.2}/-\alpha^{4.2}$、$\beta^{CD41-42}/\beta^{N}$	1	0.14

$-\alpha^{4.2}/-\alpha^{4.2}$、$\beta^{CD17}/\beta^{N}$	1	0.14
$-\alpha^{4.2}/--^{SEA}$、$\beta^{IVS\text{-}I\text{-}1}/\beta^{N}$	1	0.14
$-\alpha^{4.2}/--^{SEA}$、$\beta^{IVS\text{-}II\text{-}654}/\beta^{N}$	1	0.14
$-\alpha^{4.2}/--^{SEA}$、$\beta^{CD41\text{-}42}/\beta^{N}$	1	0.14
$-\alpha^{3.7}/\alpha\alpha$、$\beta^{IVS\text{-}II\text{-}654}/\beta^{-28}$	1	0.14
$-\alpha^{3.7}/\alpha\alpha$、$\beta^{IVS\text{-}II\text{-}654}/\beta^{CD17}$	1	0.14
$-\alpha^{3.7}/-\alpha^{4.2}$、$\beta^{CD41\text{-}42}/\beta^{N}$	1	0.14
$-\alpha^{3.7}/-\alpha^{4.2}$、$\beta^{CD17}/\beta N$	1	0.14
$-\alpha^{3.7}/-\alpha^{3.7}$、$\beta^{CD41\text{-}42}/\beta^{N}$	1	0.14
$-\alpha^{3.7}/--^{SEA}$、β^{CD26}/β^{N}	1	0.14
$-\alpha^{3.7}/--^{SEA}$、$\beta^{CD71\text{-}72}/\beta^{N}$	1	0.14
Total	699	100.00

α, normal production of the α-globin polypeptide chain; α^0, no production of the α-globin polypeptide chain; α^+, reduced production of the α-globin polypeptide chain; β^N, normal production of the β-globin polypeptide chain; β^0, no production of the β-globin polypeptide chain; β^+, impaired production of the β-globin polypeptide chain.
